# Experimental investigation of a powered lifting assistance device introducing direct touch between the caregiver and the care receiver

**DOI:** 10.3389/fbioe.2025.1556501

**Published:** 2025-03-11

**Authors:** Mari Kurata, Ming Jiang, Kotaro Hoshiba, Yusuke Sugahara, Takahiro Uehara, Masato Kawabata, Ken Harada, Yukio Takeda

**Affiliations:** ^1^ Department of Mechanical Engineering, Institute of Science Tokyo, Meguro-ku, Tokyo, Japan; ^2^ Hirakata General Hospital for Developmental Disorders, Hirakata-shi, Osaka, Japan

**Keywords:** assistive device, transfer, lifting assistance, human cooperating system, intention detection, physical therapists, direct touch

## Abstract

**Introduction:**

Transferring a patient from one place to another is one of the most strenuous works in nursing care. To address this issue, we proposed a concept for a lifting assistance device that uses two cables to perform operations such as translation, rotation, and stay. It facilitates direct touch between the caregiver and the care receiver, allowing intuitive adjustments of position and posture based on the caregiver’s intention, detected through variations in cable tension.

**Methods:**

To investigate the effectiveness of this concept, lifting experiments using a fabricated prototype were conducted. Twelve subjects, including four physical therapists (PTs) and eight subjects having no transfer experience, acted as caregivers, and a dummy was used as the care receiver.

**Results:**

Results show that regardless of the transfer experience, the caregiver’s intention detection and adjustment of the care receiver’s position and posture were successfully achieved with an accuracy of over 70%.

**Discussion:**

Survey feedback collected after the lifting experiments confirmed that utilizing direct touch between the caregiver and the care receiver was highly valued by all subjects, with a 5-point Likert scale rating both PTs (average score: 4.8 points) and non-experienced subjects (average score: 4.3 points).

## 1 Introduction

Nursing care, especially tasks that involve lifting heavy weights, presents significant challenges in ensuring the safety and wellbeing of caregivers and care receivers (Charney and Hudson, 2003). It is reported that 84.2% of caregivers have experienced low back pain (Ovayolu et al., 2014), underscoring the physical burden of their duties. This concern is exacerbated by a critical shortage in the nursing workforce (Nardi and Gyurko, 2013), a situation aggravated by the global increase in the aging population.

Among various nursing tasks, manually transferring care receivers from one place to another, such as from a bed to a wheelchair, requires high physical demands for caregivers (Banks et al., 2024). A primary concern is the minimization of physical burden to mitigate the risk of low back pain. In response to these challenges, the No Lift Policy has been promoted by organizations such as the UK Royal College of Nursing and the Australian Nurses Federation since the 1990s, which advocated for the use of appropriate transfer assistance devices instead of manual lifting by caregivers (Royal College of Nursing, 2000; Australian Nurses Federation Victoria Branch, 1998). This policy has gained widespread acceptance globally, promoting the development and utilization of various transfer assistance devices and tools.

Ensuring a safe and comfortable transfer directly impacts the quality of life of both caregivers and care receivers (Mengyuan et al., 2015; Abdelmoneium et al., 2017; Liu et al., 2020). Selecting the appropriate methods, devices, and tools for transfer is a critical decision that should address the needs of both caregivers and care receivers. The Occupational Safety and Health Administration proposes guidelines outlining four factors for examining the condition of care receivers: the required level of assistance, the body size and weight, the ability and willingness to understand and cooperate, and their medical conditions (U.S. Department of Labor and Occupational Safety and Health Administration, 2003). The most challenging scenario involves transferring care receivers with severe physical conditions who cannot bear their weight, are not cooperative, or lack upper extremity strength. Transferring a care receiver from a bed to a chair requires two caregivers to lift the care receiver’s entire body, along with the use of a sling lift (U.S. Department of Labor and Occupational Safety and Health Administration, 2003).

Based on these guidelines and diverse needs, various types of transfer assistance devices have been developed to support caregivers (Orun et al., 2015). For one of the most frequent transfer procedures—moving the care receiver from a bed to a wheelchair—assistance devices can be roughly categorized into three types, corresponding to the initial and final postures of the care receiver: devices for standing during the transfer process (Tang et al., 2018; Goh et al., 2014), devices for maintaining the care receivers in a sitting position during transfer (Grevelding and Bohannon, 2001; Wu and Shino, 2021; Johnson and Bostelman, 2010), and devices for transferring patients from a supine to a sitting position. These include lift types, including ceiling lifts and floor lifts (Marras et al., 2009), and transforming bed types (Peng et al., 2010). Regardless of the device type, powered assistive devices have been proven to be effective in reducing the caregiver’s burden, particularly in terms of waist strain and lower back pain (Vinstrup et al., 2020; Andersen et al., 2014; Zhuang et al., 1999; Abdul Halim et al., 2023). The feature of powered assistive devices further motivates the development of innovative robotic devices, such as RIBA (Ming et al., 2014), Smart Hoist (Ranasinghe et al., 2014), robotic-assisted transfer device (RATD) (Burkman et al., 2017), and the AgileLife Patient Transfer System (Kulich et al., 2023).

In this study, the focus is on the process of transferring patients with severe physical conditions from a supine to a sitting position. Lift types, including ceiling lifts and floor lifts, are the most widely used (Kucera et al., 2019). The advantages of lifts are they are easy to use, have reasonable prices compared to robotic devices, and can be shared among the care receivers (Krishnan and Pugazhenthi, 2014). Furthermore, the difference between lift types and transforming bed types is that lift-type devices can prevent the care receivers from developing symptoms of pressure ulcers since the care receiver’s entire body completely leaves the bed (Sivaprakasam et al., 2017). Furthermore, changing the care receiver’s posture and vision during transfer effectively enhances the care receiver’s psychological awareness and health (Sivaprakasam et al., 2017; Fuchs and Koch, 2014).

Despite the widespread availability of transfer assistance devices, their application in nursing facilities remains limited (Kucera et al., 2019). According to Vinstrup et al. (2020), in a total of 540 full patient transfers and use of 14 different assistive devices, 53% of patient transfers were conducted without any assistive devices. The reluctance to use transfer assistive devices can be attributed to the complexities and concerns for patient safety involved in accommodating the needs of care receivers, particularly those in severe physical conditions (Garg and Kapellusch, 2012). In conventional lifts, the patient’s posture is uniquely fixed because the cables of the sling seat are converged at a single point of suspension, which may result in insufficient trunk and cervical spine support. This inadequacy has been shown to increase the risk of injury and cultivate feelings of insecurity for both caregivers and care receivers (Luz and Echternacht, 2012). The sensation of being suspended in the air by a sling seat can cause the patients to sway or rotate. This instability, along with mental anxiety, may pose challenges for care receivers with cognitive impairments, who may exhibit sudden movements, resistance, or aggression, thereby risking balance loss (Fuchs and Koch, 2014; Miller et al., 2006). These problems rarely occur when the care receiver is transferred manually by two caregivers, so the use of lift-type devices is still limited because some caregivers or care receivers prefer not to use any devices. This situation underscores the need for developing transfer-assisting devices that not only relieve the burden of caregivers but also include careful considerations of providing a secure, comfortable experience for care receivers.

To address these challenges, we propose a novel lifting assistance device that integrates direct touch between caregivers and care receivers, combining the benefits of powered and manual lifts. This direct touch feature is designed to enhance psychological comfort, fostering a sense of safety and relief for both caregivers and care receivers (Koji, 2018; Routasalo and Isola, 1996). To simulate caregiving for a severely impaired patient unable to support their own weight, we conducted experiments using a dummy. Unlike conventional lifts, which rely on single-point suspension, our device employs two cables, which are attached near the neck and hip joints of the care receiver. This configuration provides enhanced support for the trunk and cervical spine while enabling adjustments to the care receiver’s position and posture. By detecting variations in cable tension caused by the caregiver’s applied force, the device aligns its movements with the caregiver’s intentions during the lifting process. Although the conceptual design and preliminary operation of the device have been introduced by Mari et al. (2023), this study focuses on validating its effectiveness and evaluating the user experience across caregivers with different levels of expertise in transfer. Additionally, we detail the criteria developed for detecting caregiver intentions and present insights into user experience obtained through experiments and surveys.

## 2 Materials and methods

To evaluate the effectiveness of the lifting assistance device in detecting caregivers’ intentions and assessing its user experience among caregivers with different levels of expertise, two series of experiments were designed and conducted. First, in the threshold determination experiments, we collected the data on cable tensions for setting thresholds at different conditions of dummy’s postures. Then, the collected cable tensions were used to fit a line with the motor rotational degrees. The slopes and intercepts of these linear relationships were then applied to different criteria for detecting the intentions. Next, the lifting experiments were conducted with thresholds determined in each criterion to evaluate the alignment of the detected intention in timing with the applied intention of caregivers. At last, surveys, including questionnaires and interviews, were conducted with the subjects to evaluate the user experience with the lifting assistance device.

The intention detection algorithm affected the user experience of the caregivers during the device’s operation. Therefore, in this study, various criteria, coded as AA, A, B, and C, were adopted to set the thresholds of cable tensions. The overview of the criteria is shown in Table 1. Twelve subjects, including four physical therapists (PTs) and eight subjects without experience in transfer (referred to as non-experienced subjects), were divided into three groups. For subjects in Group I and Group III, both the threshold determination experiments and the lifting experiments were conducted. For subjects in Group II, only the lifting experiments were conducted. Criteria A, B, and C used for Group II in the lifting experiments were determined based on the cable tension data collected from Group I. Criterion AA was the individual criterion decided based on the collected cable tension data on each subject in groups I and III. Criteria A, B, and C were the common criteria formed with different formulas applied to Group II, which were determined based on the cable tension data on subjects in Group I. The individual criterion AA and common criteria A, B, and C were set to investigate the insights for further application in real scenarios.

**TABLE 1:** Overview of criteria for different experiments.

Subject group	Transfer experience	Threshold determination experiment	Lifting experiment			Questionnaire and interview
Group I	None	Yes	AA			Yes
Group II	None	No	A	B	C	Yes
Group III	Yes (physical therapists)	Yes	AA			Yes

### 2.1 Concept and design of the lifting assistance device

**FIGURE 1:**
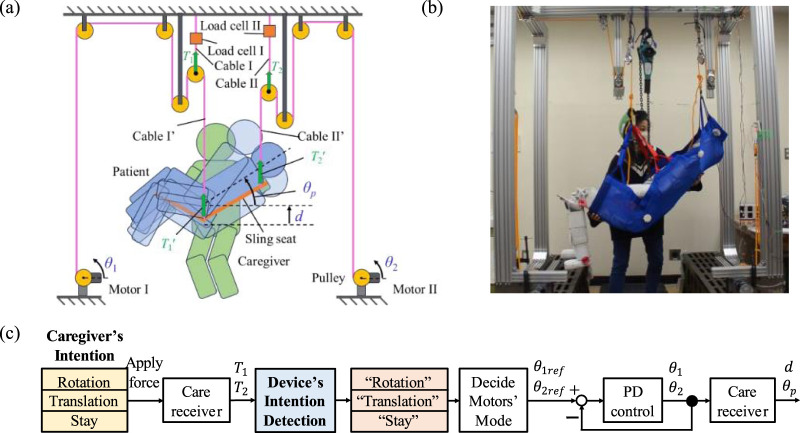
Structure and operation flow of the proposed device. **(A)** Structure of the proposed lifting assistance device. **(B)** Fabricated lifting assistance device. **(C)** Intention detection flow.

The structure of the lifting assistance device and its components are illustrated in Figures 1A, B. The device incorporates a sling seat positioned beneath the care receiver, from which two cables extend to support the care receiver’s entire body. Cable I′, attached near the care receiver’s hip joint, and Cable II’, attached near the care receiver’s neck, are pivotal for the lifting mechanism. During the lifting process, the caregiver maintains direct touch with the care receiver at the backside of the shoulders and knees with both arms.

The device executes three fundamental movements to adjust the care receiver’s position and posture: rotation, where the care receiver’s upper body angle $\theta_{p}$ increases; translation, where the care receiver’s entire body position d elevates; and stay, where both $\theta_{p}$ and d remain unchanged. Accordingly, the caregiver’s intentions during the lifting process are categorized into rotation, translation, and stay.

The operation flow of the device is shown in Figure 1C. When the caregiver intends to change the care receiver’s position or posture, the applied force on the care receiver causes variations in cable tensions $T_{1}$ ’ and $T_{2}$ ’ for cables I’ and II’, respectively. To measure the tensions within cables I’ and II’, two movable pulleys and load cells (Load Cell I and II) are attached to Cable I and II. These load cells measure the tension values $T_{1}$ and $T_{2}$ . In this way, $T_{1}$ ’ and $T_{2}$ ’ can be described by $T_{1}=2T_{1}\prime ,T_{2}=2T_{2}\prime \;.$ The adjustment of the care receiver’s position d and posture $\theta_{p}$ can be achieved by manipulating the angles $\theta_{1}$ and $\theta_{2}$ of motors I and II, which control the winding of cables I’ and II’, respectively. Once the caregiver’s intention is detected by the device, the lengths of the two cables adjust according to the reference positions $\theta_{1ref}$ and $\theta_{2ref}$ of the two motors. For the rotation intention, only Motor II runs to elevate the care receiver’s upper body. For the translation intention, both motors I and II run to lift the care receiver’s entire body. For the stay intention, both motors pause to maintain the care receiver’s current position and posture.

### 2.2 Subjects’ information

Twelve subjects were categorized into three groups based on their experience with transfer. Group I (subjects #1–4, 171 ± 9 cm in height) and Group II (subjects #5–8, 177 ± 7 cm in height) each had four male subjects. All eight subjects were aged between 20 and 30 years and had no prior experience in transfer assistance. Group III (subjects #9–12, 168 ± 3 cm in height) included four male physical therapists (PTs) aged 20–50 years, all with extensive experience in transfer assistance.

In this study, all subjects were assigned to play the role of a caregiver. The dummy was used as a care receiver with severe disabilities. The dummy measured 165 cm in height and weighed 43 kg.

All experiments in this study were conducted following the Ethical Guidelines for Life Science and Medical Research Involving Human Subjects. The protocol for this study received approval from the Ethics Review Committee of the Institute of Science Tokyo, approval number 2022251.

### 2.3 Threshold determination experiments

First, the threshold determination experiments were conducted to collect data on tension variation in cables corresponding to various postures of the dummy based on the subjects’ intended actions. Subjects in groups I and III were instructed to continuously apply force to the dummy in alignment with one of three intentions: stay, rotation, or translation, as indicated by the LED signals. Load cells I and II continuously measured the cable tensions across different upper body postures of the dummy. Each subject performed the lifting operation three times, with each trial lasting 40 s.

During data collection, Motor I remained off, while Motor II operated at a slow and constant speed to gradually adjust the dummy’s upper body posture $\theta_{p}$ from 40 to 70°. This gradual adjustment allowed for the systematic collection of cable tension data across various postures and three intention conditions. Since the dummy was heavy, operating the motor at a slow speed ensured that the subjects were not subjected to physical burden during the experiments, minimizing the risk of fatigue or injury.

### 2.4 Criteria for detecting intentions

Since the criteria setting related to the movement of the lifting assistance device affected the user experience, we designed and evaluated various criteria to detect the caregivers’ intentions. For our device, operational user experience was primarily influenced by three key factors: the formulas of the tension values used to distinguish intentions, the coefficient values of the formulas derived from the threshold determination experiments, and the motor speed affecting how quickly the device moves. In this study, formulas and coefficients varied, and the motor speed remained constant across all experiments.

Criterion AA was designed to vary the coefficients, while criteria A, B, and C were designed to vary the formulas. Criterion AA defined the thresholds based on individual results from the threshold determination experiments, allowing for personalized coefficients used in the subsequent lifting experiments. Criterion AA was applied to groups I and III. Criteria A, B, and C defined the thresholds for Group II, which were determined by averaging the results from four subjects in Group I during the same experiment, offering common thresholds for the lifting experiments.

#### 2.4.1 Criterion AA

Criterion AA is determined based on the sum (*T*_1_+*T*_2_) and the ratio of the tension values ((*T*_2_/*T*_1_+*T*_2_) × 100) in the cables. AA-I for the sum is defined in Equation 1 to distinguish the stay intention from the others.

AA-I

$$\begin{array}{c} T_{1}+T_{2}\leq \frac{\left(p_{T,rot,\#XX}+p_{T,trans,\#XX}\right)+2p_{T,stay,\#XX}}{4}\cdot \left(\theta_{2}-\theta_{1}\right)+\\ \frac{\left(q_{T,rot,\#XX}+q_{T,trans,\#XX}\right)+2q_{T,stay,\#XX}}{4} \end{array}$$ (1)

Criterion AA-II for the ratio of tension values in Equation 2 is defined to differentiate between rotation and translation intentions.

AA-II

$$\begin{array}{c} \frac{T_{2}}{T_{1}+T_{2}}\cdot 100\leq \frac{\left(p_{R,rot,\#XX}+p_{R,trans,\#XX}\right)}{2}\cdot \left(\theta_{2}-\theta_{1}\right)+\frac{\left(q_{R,rot,\#XX}+q_{R,trans,\#XX}\right)}{2} \end{array}$$ (2)

In these equations, *p* and *q*, respectively, represent the slopes and intercepts of the fitted straight lines of tension values relative to motor angle (Figure 3), which were obtained from each subject’s data on stay, rotation, and translation intentions. The subscripts *T* and *R* denote the total tension (*T*_1_+*T*_2_) and the ratio of cable tension ((*T*_2_/*T*_1_+*T*_2_) × 100), respectively. *stay*, *rot*, and *trans* correspond to the data on stay, rotation, and translation intentions, respectively. *#XX* refers to the subject’s code, specifying these parameters to each subject’s data, which were obtained during the threshold determination experiment. For example, $p_{T,stay,\#1}$ represents the slope value of the fitting straight-line obtained from the tension data on subject #1 with the stay intention relative to motor angles. If the tension values measured in the lifting experiment do not meet the criterion AA-I, the device interprets the caregiver’s intention as stay. If the tension value meets criterion AA-I, then criterion AA-II is examined. If the tension ratio meets AA-I and AA-II at the same time, the intention is interpreted as rotation; if only AA-I is met, the detected intention is classified as translation.

#### 2.4.2 Criteria A, B, and C

Since the lifting assistance device is expected to be used by multiple caregivers in real-world scenarios, it is convenient to apply a common threshold across different users. Therefore, criteria A, B, and C were designed using the average slopes and intercepts from the data on the four subjects in Group I.

Criterion A was obtained by averaging the slopes and intercepts derived from criteria AA-I and AA-II for subjects #1–4 in Group I. A-I corresponds to the total tension (*T*_1_+*T*_2_), while A-II represents the tension ratio ((*T*_2_/*T*_1_+*T*_2_) × 100). Similar to the decision process used for criteria AA-I and AA-II, criterion A-I is formulated to distinguish the stay intention from others and is expressed as shown in Equation 3:

A-I

$$\begin{array}{c} T_{1}+T_{2}\leq \sum_{XX=1}^{4}\frac{\left(p_{T,rot,\#XX}+p_{T,trans,\#XX}\right)+2p_{T,stay,\#XX}}{4}\cdot \left(\theta_{2}-\theta_{1}\right)+\\ \sum_{XX=1}^{4}\frac{\left(q_{T,rot,\#XX}+q_{T,trans,\#XX}\right)+2q_{T,stay,\#XX}}{4} \end{array}$$ (3)

A-II distinguishes between rotation and translation intentions and is defined as shown in Equation 4:

A-II

$$\begin{array}{c} \frac{T_{2}}{T_{1}+T_{2}}\cdot 100\leq \sum_{XX=1}^{4}\frac{\left(p_{R,rot,\#XX}+p_{R,trans,\#XX}\right)}{2}\cdot \left(\theta_{2}-\theta_{1}\right)+\\ \sum_{XX=1}^{4}\frac{\left(q_{R,rot,\#XX}+q_{R,trans,\#XX}\right)}{2} \end{array}$$ (4)

Criterion B utilizes the product $\left(T_{1}\cdot T_{2}\right)$ and the difference ( $T_{1}-T_{2}$ ) values of cable tensions, as shown in Equations 5, 6. B-I discriminates between stay and the other two intentions.

B-I

$$\begin{array}{c} T_{1}\cdot T_{2}\leq \sum_{XX=1}^{4}\frac{\left(p_{M,rot,\#XX}+p_{M,trans,\#XX}\right)+2p_{M,stay,\#XX}}{4}\cdot \left(\theta_{2}-\theta_{1}\right)\\ +\sum_{XX=1}^{4}\frac{\left(q_{M,rot,\#XX}+q_{M,trans,\#XX}\right)+2q_{M,stay,\#XX}}{4} \end{array}$$ (5)

B-II distinguishes between rotation and translation:

B-II

$$\begin{array}{c} T_{1}-T_{2}\leq \sum_{XX=1}^{4}\frac{\left(p_{S,rot,\#XX}+p_{S,trans,\#XX}\right)}{2}\cdot \left(\theta_{2}-\theta_{1}\right)+\sum_{XX=1}^{4}\frac{\left(q_{S,rot,\#XX}+q_{S,trans,\#XX}\right)}{2} \end{array}$$ (6)

The subscripts *M* and *S* indicate the data derived from the product $\left(T_{1}\cdot T_{2}\right)$ and the difference ( $T_{1}-T_{2}$ ) of the cable tensions, respectively. Criterion B allows the device to classify the caregiver’s intention as stay when the product value does not satisfy B-I. If the tension value satisfies both B-I and B-II simultaneously, the intention is identified as translation. If only B-I is satisfied, the intention is interpreted as rotation.

Criterion C is designed based on the changes in tension values $T_{1}$ and $T_{2}$ between two consecutive intended movements. Criterion C employs deviation values $∆T_{1}\left(t\right)$ and $∆T_{2}\left(t\right)$ , as shown in Equations 7, 8, which are defined as the difference between the current tension values at time t [s] and those measured at a previous time t−10⋅∆t [s], where ∆t represents the sampling period of 50 ms:

$$∆T_{1}\left(t\right)=T_{1}\left(t\right)-T_{1}\left(t-10\cdot ∆t\;\right),$$ (7)

$$∆T_{2}\left(t\right)=T_{2}\left(t\right)-T_{2}\left(t-10\cdot ∆t\;\right).$$ (8)

Criterion C (C-I to C-IV) is defined as shown in Equations 9–12, respectively, where $a_{D1}$ , $a_{D2}$ , $b_{D1}$ , and $b_{D2}$ are the upper and lower limits:

C-I

$$-b_{D1}\leq ∆T_{1}\left(t\right)\leq -a_{D1}.$$ (9)

C-II

$$a_{D1}\leq ∆T_{1}\left(t\right)\leq b_{D1}.$$ (10)

C-III

$$-b_{D2}\leq ∆T_{2}\left(t\right)\leq -a_{D2}.$$ (11)

C-IV

$$a_{D2}\leq ∆T_{2}\left(t\right)\leq b_{D2}.$$ (12)

**FIGURE 2:**
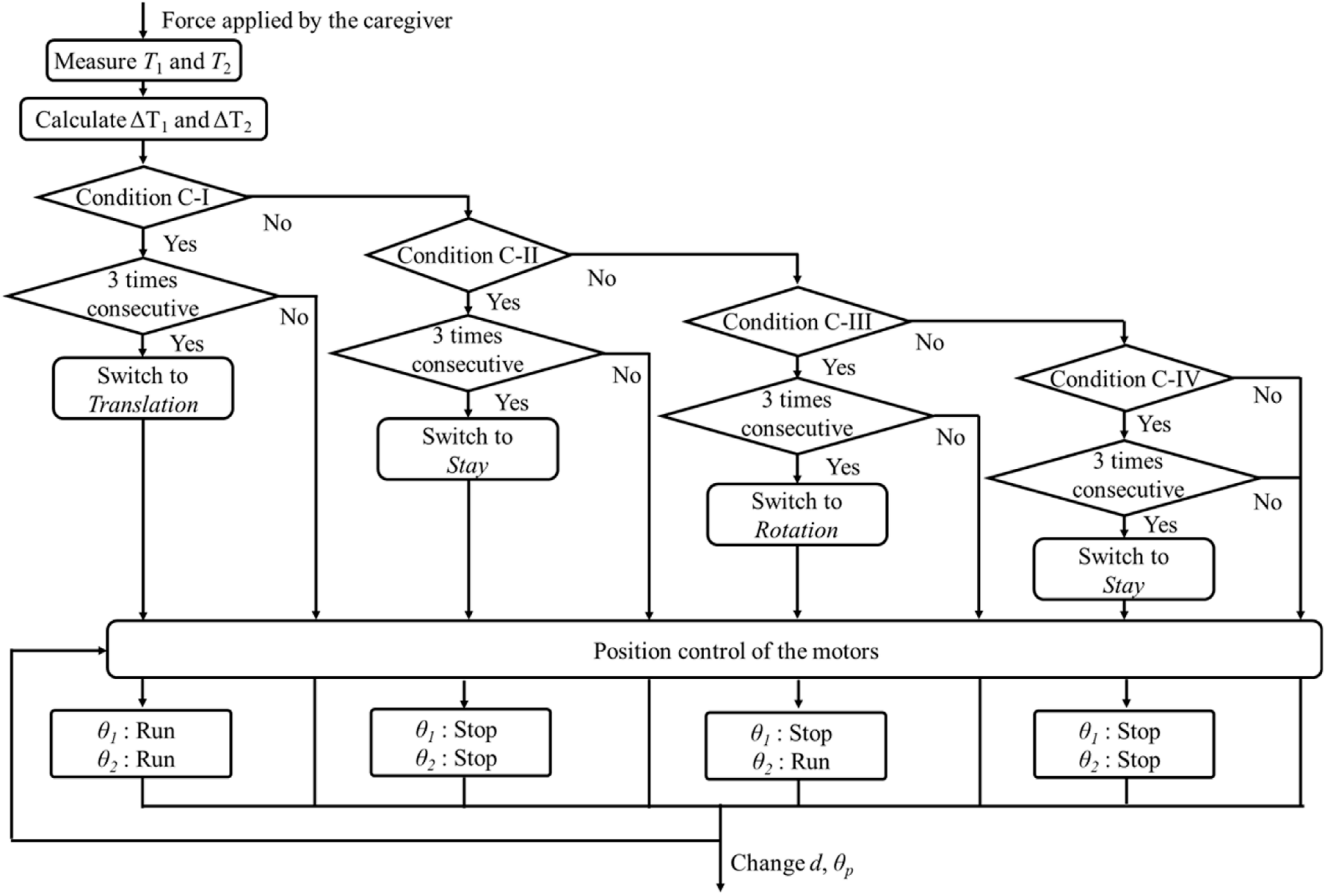
Intention detection flow for criterion C.

The intention detection flow for criterion C is illustrated in Figure 2. The device first identifies the measured change in cable tension and then evaluates criteria C-I, C-II, C-III, and C-IV sequentially. For instance, in criterion C-I, the judgment process is repeated three consecutive times. The lifting assistance device will execute the translation movement only if the translation intention is detected three times in succession; otherwise, criterion C-II is evaluated. The evaluation process for criteria C-II, C-III, and C-IV follows the same procedure.

### 2.5 Lifting experiments

Lifting experiments were conducted after applying one of the criteria shown in Table 1. During the experiments, subjects were instructed to apply forces to the dummy according to the intentions indicated by LEDs in the following sequence: rotation → stay → translation → stay. This sequence was repeated for three sets, with each intention lasting 6 seconds. In response to the detected intentions, motors I and II operated to adjust the dummy’s hip joint position d and upper body posture $\theta_{p}$ , respectively. The effectiveness of the proposed intention detection flow for each criterion was evaluated based on the consistency rate between the timing of the intentions indicated by the LEDs and those detected by the device.

### 2.6 User experience surveys

After completing the lifting experiments, subjects were asked to fill out a questionnaire to evaluate their experience with the lifting assistance device. The questionnaire, shown in Table 2, utilized a 5-point Likert scale to assess various aspects of the device’s operation and its impact on the user through eight questions.

**TABLE 2:** Contents of the questionnaire.

Q1. Fatigue	Did you feel tired when applying force to the dummy? If yes, please indicate the body parts on the physical chart.
Q2. Pain	Did you experience any pain when applying force to the dummy? If yes, please indicate the painful body parts on the physical chart.
Q3. Dummy stability	How confident were you that the dummy would remain stable and not fall during the experiment?
Q4. Motion alignment	Do you think the dummy’s position and posture were adjusted according to your expectations?
Q5. Device compliance	Do you think the device responded accurately to your intentions and operations?
Q6. Operation method	How easy was it to understand and execute the method of applying force to the dummy based on your intended actions?
Q7. Conversation	Do you think it is feasible to engage in conversation with the dummy (care receiver) while operating the device?
Q8. Direct touch	Do you think the direct physical touch with the dummy (care receiver) enhances your sense of security or relief? For example, when compared with using a remote controller?

Q1 and Q2 focused on the user’s physical experience, especially fatigue or pain encountered while operating the device. Subjects were also instructed to indicate any affected body parts using physical charts.

Q3 to Q5 explored the technical aspects of the device, including the effectiveness of the cable tension settings, the appropriateness of threshold parameters, and the responsiveness of the motors.

Q6 to Q8 addressed the overarching concept of the proposed device, seeking feedback on its design philosophy, user-friendliness, and potential impact on the caregiving process.

## 3 Results

### 3.1 Threshold determination experiments

#### 3.1.1 Criterion AA for groups I and III

The threshold determination experiments, as described in Section 2.3, were conducted with eight subjects from groups I and III.

**FIGURE 3:**
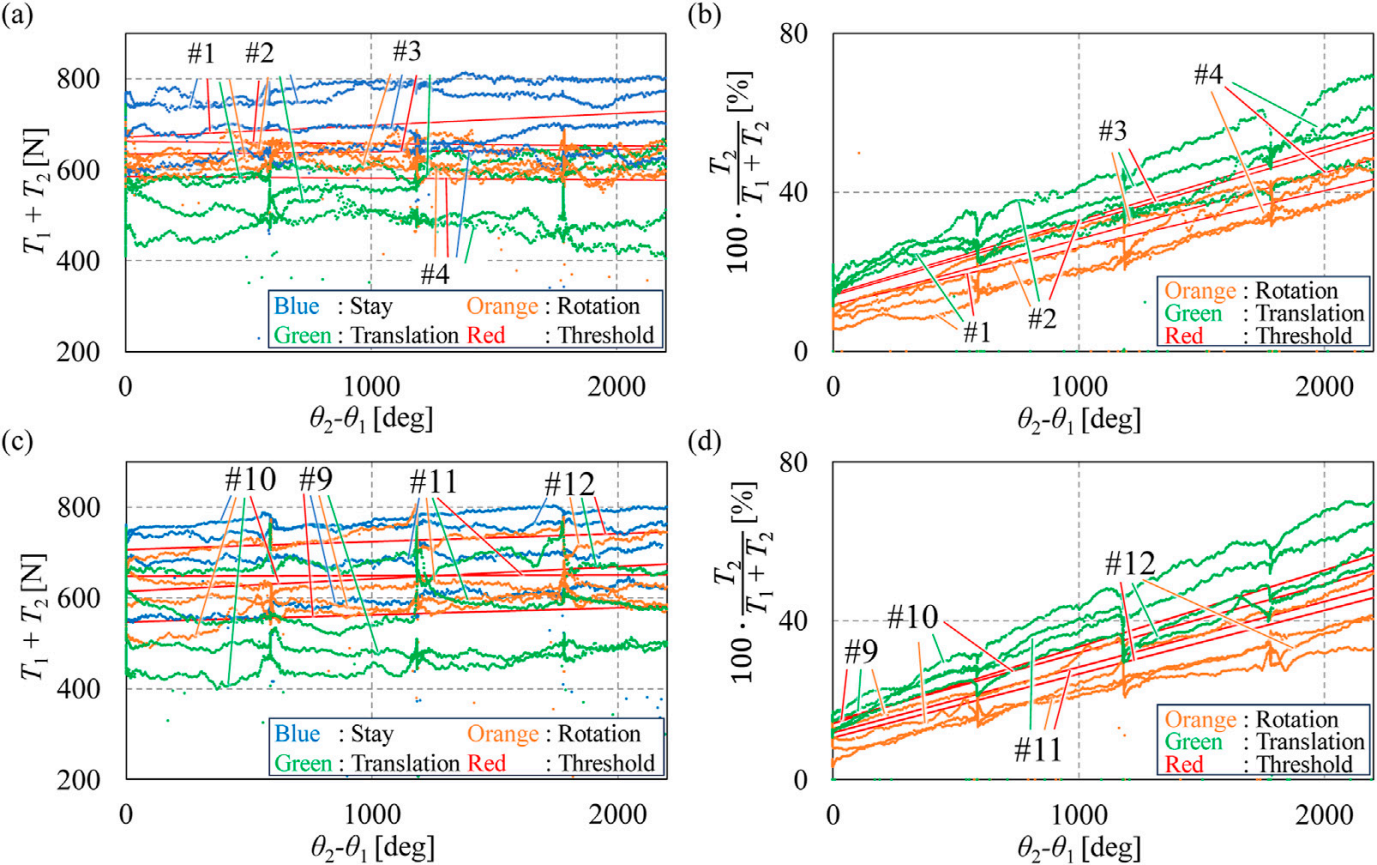
Tension values in threshold determination experiments with groups I and III. **(A)** Values of $T_{1}+T_{2}$ and fitted straight lines of Group I. **(B)** Values of $T_{2}/\left(T_{1}+T_{2}\right)$ and fitted straight lines of Group I. **(C)** Values of $T_{1}+T_{2}$ and fitted straight lines of Group III. **(D)** Values of $T_{2}/\left(T_{1}+T_{2}\right)$ and fitted straight lines of Group III.

Figures 3A, B display the data from Group I, used to define criteria AA-I, AA-II, A-I, and A-II, while Figures 3C, D present the data on Group III used for defining criteria AA-I and AA-II. The data from the eight subjects exhibited a linear relationship between the horizontal and vertical axes. The slopes and intercepts of the red-fitted straight line were used to determine the thresholds. In all figures shown in Figure 3, the total tension (*T*_1_+*T*_2_) and the ratio of cable tension ((*T*_2_/*T*_1_+*T*_2_) × 100) can be observed across the three intentions, which are represented in blue, orange, and green. Criterion AA-I, with thresholds indicated in red in Figures 3A, C, distinguishes the stay intention from the other two. Similarly, criterion AA-II, with thresholds shown in red in Figures 3B, D, differentiates between the rotation and translation intentions.

**FIGURE 4:**
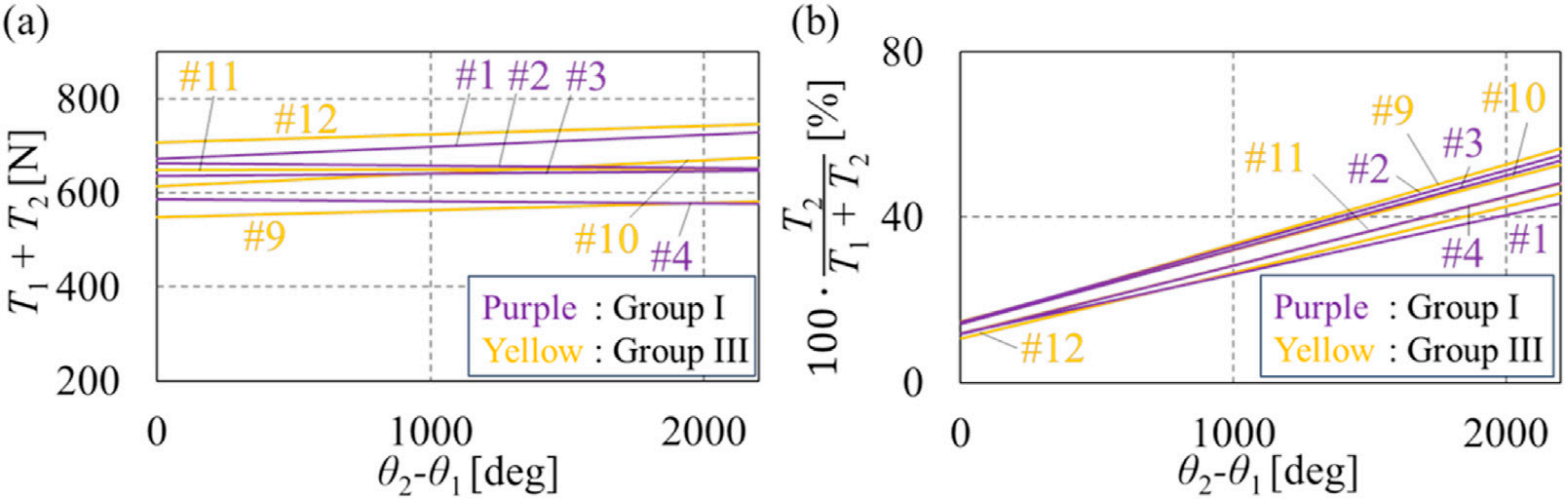
Summarized fitted straight lines of groups I and III. **(A)** Summarized fitted straight lines of tension sum. **(B)** Summarized fitted straight lines of the tension ratio.

Figure 4 presents the fitted straight lines for all eight subjects, with purple indicating the results from Group I and yellow representing those from Group III. A *t*-test was conducted on the slopes and intercepts of the lines shown in Figure 4. The results indicate that there is no significant difference in the slopes and intercepts of the fitted straight lines between subjects in groups I and III.

#### 3.1.2 Criteria A, B, and C for Group II

**FIGURE 5:**
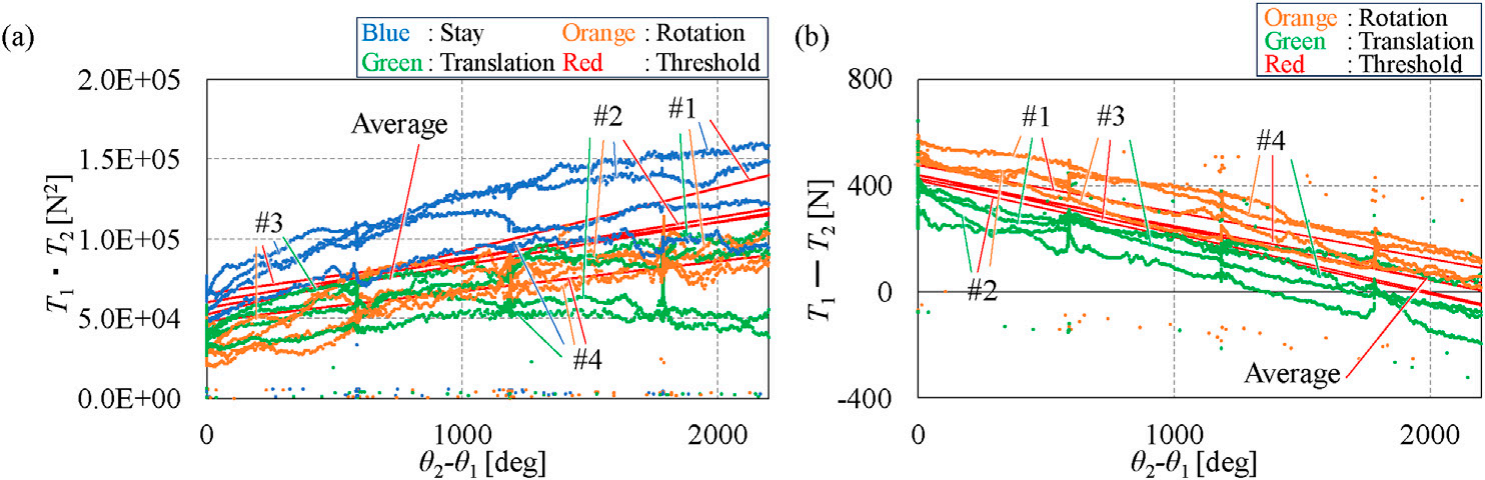
Data of Group I used for defining criterion B. **(A)** Values of $T_{1}\cdot T_{2}$ and fitted straight lines. **(B)** Values of $T_{1}-T_{2}$ and fitted straight lines.

Criteria A, B, and C serve as common criteria for all subjects in Group II. Criteria A and B were defined using the average tension data from four subjects in Group I. Criterion A was derived from the average slopes and intercepts of the fitted straight lines in Figures 3A, B. The average slope and intercept values for criterion A-I are 0.00525 and 639, respectively, while for A-II, they are 0.017 and 13.0, respectively. Criterion B was formulated using the average slopes and intercepts shown in Figures 5A, B, with a slope value of 27.0 and an intercept value of 56,600 for B-I and a slope value of −0.20 and an intercept value of 441 for B-II. By substituting these slope and intercept values into Equations 3, 6, criteria A and B were obtained as shown in Equations 13–16, respectively:

A-I

$$T_{1}+T_{2}\leq 5.25\times 10^{-3}\cdot \left(\theta_{2}-\theta_{1}\right)+639.$$ (13)

A-II

$$\frac{T_{2}}{T_{1}+T_{2}}\cdot 100\leq 0.017\cdot \left(\theta_{2}-\theta_{1}\right)+13.0.$$ (14)

B-I

$$T_{1}\cdot T_{2}\leq 27.0\cdot \left(\theta_{2}-\theta_{1}\right)+5.66\times 10^{4}.$$ (15)

B-II

$$T_{1}-T_{2}\leq -0.20\cdot \left(\theta_{2}-\theta_{1}\right)+441.$$ (16)

**FIGURE 6:**
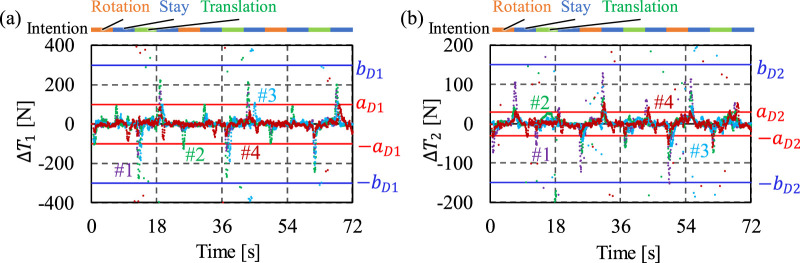
$∆T_{1}$ and $∆T_{2}$ in the threshold determination experiments. **(A)** Coefficient set for criteria C-I and C-II. **(B)** Coefficients set for criteria C-III and C-IV.

The coefficients for criterion C were determined based on $∆T_{1}$ and $∆T_{2}$ during the threshold determination experiment, as shown in Figures 6A, B. The coefficients were set as $a_{D1}=100\;N$ , $b_{D1}=300\;N$ , $a_{D2}=30\;N$ , and $b_{D2}=150\;N$ . Therefore, criterion C can be expressed as shown in Equations 17–20, respectively:

C-I

$$-300\leq ∆T_{1}\left(t\right)\leq -100.$$ (17)

C-II

$$100\leq ∆T_{1}\left(t\right)\leq 300.$$ (18)

C-III

$$-150\leq ∆T_{2}\left(t\right)\leq -30.$$ (19)

C-IV

$$30\leq ∆T_{2}\left(t\right)\leq 150.$$ (20)

### 3.2 Lifting experiments with criterion AA using individual thresholds

Following the establishment of criterion AA and the application of individualized thresholds for each subject, lifting experiments were conducted with subjects from groups I and III.

#### 3.2.1 Consistency rate results

**FIGURE 7:**
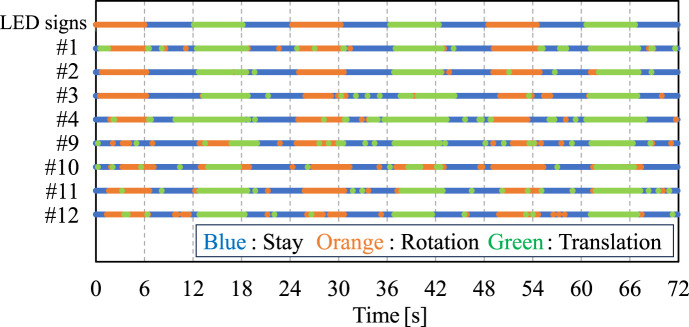
Intentions indicated by LEDs to the subject and the results of device’s intention detection for groups I and III.

**FIGURE 8:**
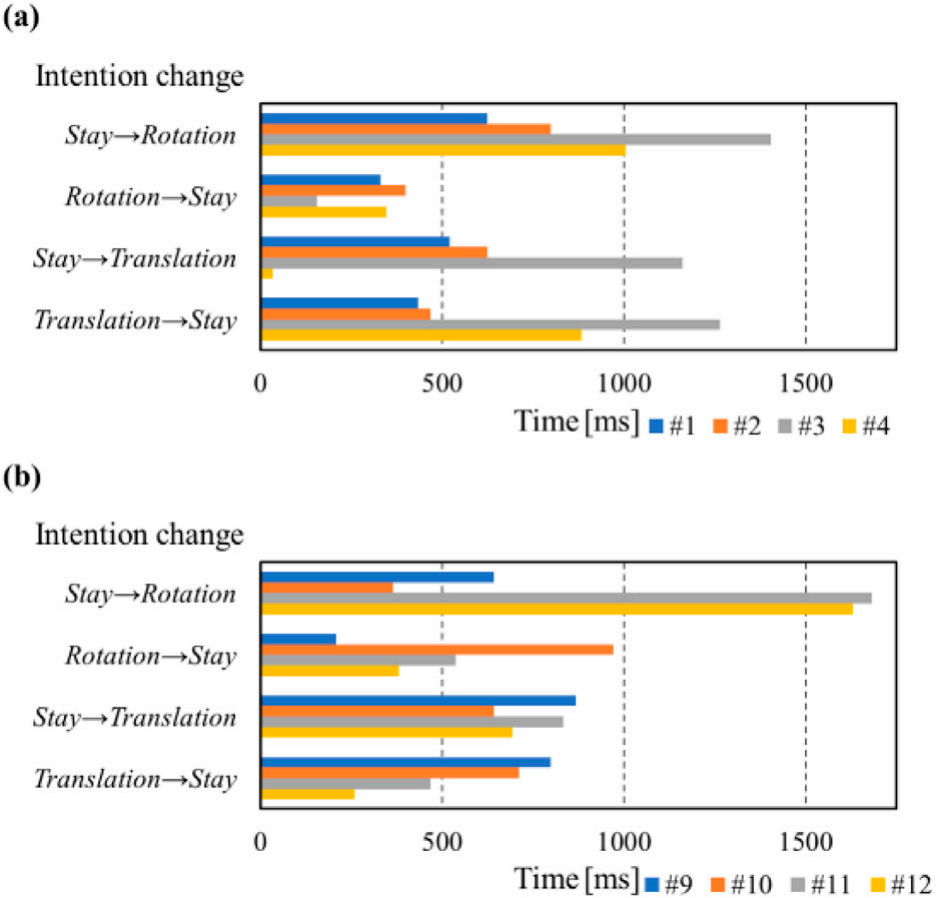
Delay time between the LED-indicated intention and the device-detected intention during each intention change period from **(A)** Group I and **(B)** Group III.

Figure 7 shows the alignment between the subject’s intentions, as signaled by LEDs, and the intentions detected by the lifting assistance device. The horizontal axis represents the experimental timeline for three sets of intention detection tests. The first line in the graph shows the LED signal timings, while the lines below display the corresponding times when each intention was detected by the device. Figure 8 illustrates the delay time between the LED-indicated intention and the device-detected intention when the subject’s intention was signaled to change by the LED.

Table 3 presents the consistency rates for Group I, which were 88%, 87%, 79%, and 81%, respectively. For Group III, the rates were 73%, 71%, 83%, and 84%, respectively. The average consistency rate was 84% for Group I and 77% for Group III. These results demonstrate the device’s capability to detect caregiver’s intentions with a high consistency ratio for both PTs and non-experienced subjects. Although PTs exhibited slightly lower consistency rates compared to non-experienced subjects, statistical analysis with the *t*-test indicates no significant difference between the two groups.

**TABLE 3:** Consistency rates of lifting experiments in each criterion and group.

	Criteria	Group I				Group I average
		#1	#2	#3	#4	
Rate [%]	AA	88.4	86.6	78.9	80.8	83.7
	Criteria	Group II				Group II average
		#5	#6	#7	#8	
Rate [%]	A	79.8	80.9	80.9	86.0	81.9
	B	85.0	86.2	75.3	81.6	82.0
	C	82.4	82.7	85.1	78.3	82.1
	Criteria	Group III				Group III average
		#9	#10	#11	#12	
Rate [%]	AA	72.8	70.6	82.5	83.9	77.4

#### 3.2.2 Motion analysis of the dummy

**FIGURE 9:**
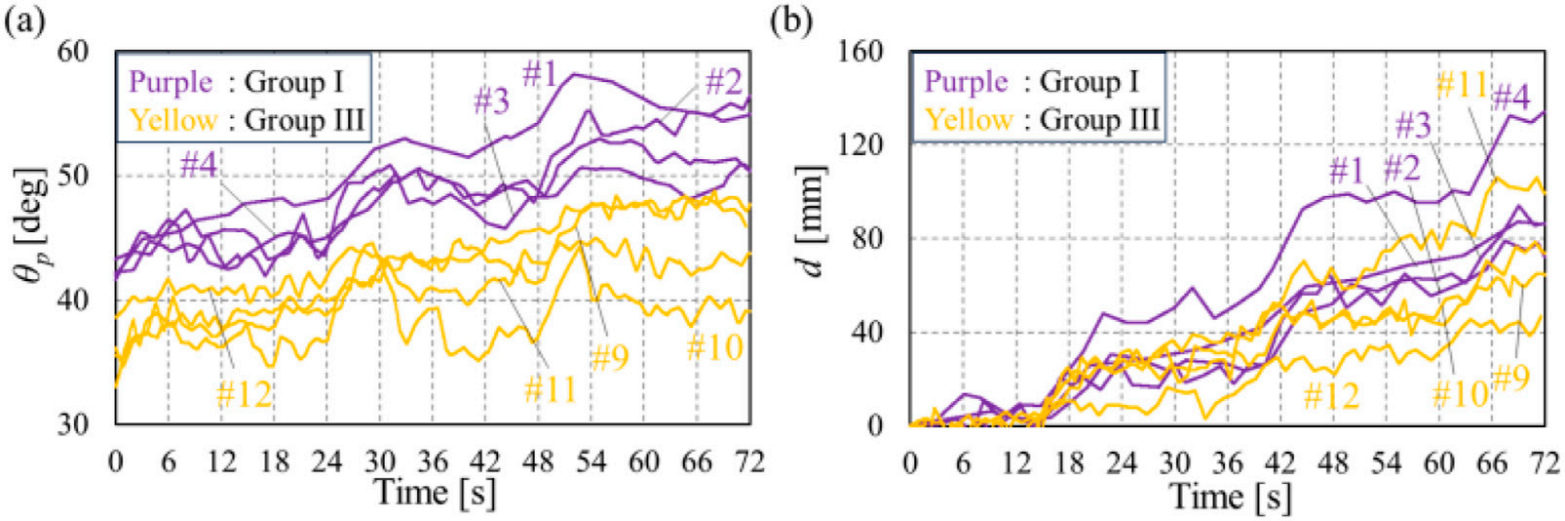
Transition of dummy’s position and posture in the lifting experiments. **(A)** Transition of *d* of groups I and III. **(B)** Transition of *θ*_p_ of groups I and III.

Figure 9 presents the measured position *d* and posture *θ*_*p*_ of the dummy during the lifting experiments, recorded using markers attached to the shoulder, hip joint, and knee of the dummy. Subjects adjusted the dummy’ position and posture following the sequence: rotation, stay, translation, and stay. In Figure 9A, during the 0–24-s period, the dummy’s position remains nearly constant, with *d* increasing between 12 and 18 s, which is consistent with the operation under translation intention. Similarly, in the same period of Figure 9B, the dummy’s upper body angle increases during the first 6 seconds and then remains nearly constant for the following 18 s, which is consistent with the operation sequence. These results demonstrate that the device successfully adjusts the position and posture of the dummy in response to the detected intention of each subject.

**FIGURE 10:**
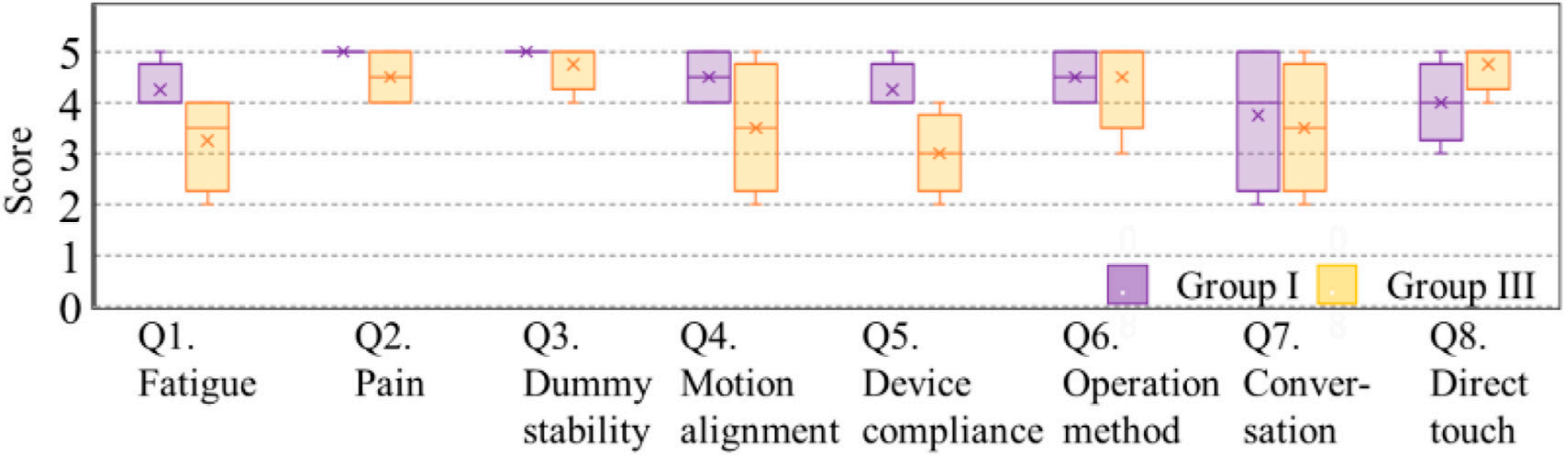
Results of 5-point evaluation in the questionnaires.

The questionnaire results are presented in Figure 10. Q2 (pain), Q3 (dummy stability), Q6 (operation method), and Q8 (direct touch) received high ratings, with average scores of 4.0 points or above from Groups I and III. In contrast, Q1 (fatigue), Q4 (motion alignment), Q5 (device compliance), and Q7 (conversation) were identified as aspects needing improvement.

### 3.3 Lifting experiments with criteria A, B, and C using common thresholds

The lifting experiments were conducted with Group II using common thresholds with criteria A, B, and C.

#### 3.3.1 Results

The consistency rates for each criterion with Group II are shown in Table 3. The *t*-test analysis results using criteria B and C showed no significant difference in consistency rates compared to criterion A, with the average consistency rate being approximately 82% across all conditions.

**FIGURE 11:**
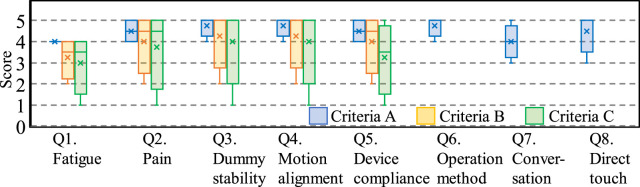
Questionnaire results of each item from Group II.

The questionnaire results from Group II, evaluating their user experience across criteria A, B, and C, are shown in Figure 11. Questions Q6 to Q8 were only answered once under criterion A as they relate to the conceptual design of the device and are independent of the applied criteria.

Compared to the questionnaire scores for criteria B and C, criterion A, which incorporates both total tension and ratio values, received the highest evaluations despite the similar consistency rates among the three criteria.

When comparing the results of criterion A in Group II and those of criterion AA in groups I and III using a *t*-test, no significant differences were observed in either the consistency rates shown in Table 3 or the questionnaire scores shown in Figures 10, 11. This observation suggests that common thresholds can be established across caregivers.

## 4 Discussion

This study proposed and evaluated a powered lifting assistance device that integrates direct touch between caregivers and care receivers to enhance the usability of the lifting assistance device. The device employs a dual-cable configuration, providing support near the hip joint and neck of the care receivers while detecting caregiver intentions through variations in cable tension. The effectiveness of the device was assessed through threshold determination experiments, lifting experiments, and user experience surveys involving both experienced PTs and non-experienced subjects.

The consistency rate, defined as the percentage of time the detected intention matches the caregiver’s actual intention, exceeded 70% across all subjects, with an average of 84% for non-experienced subjects (Group I) and 77% for physical therapists (PTs, Group III). Although non-experienced subjects exhibited slightly higher consistency rates, statistical analysis indicated no significant difference between the two groups. This finding suggests that even individuals with no prior experience in patient transfers can effectively use the intention detection method, implying its potential for broader application among caregivers.

However, the intention detection process exhibited a delay time of approximately 1 s, as shown in Figure 8. This delay is attributed to two main factors. First, the reference time of the LED: caregivers required reaction time before executing the instructed movement. Second, the device required time to detect the intention and activate the motors accordingly. Despite this, a delay of 1–2 s is considered acceptable for non-urgent, controlled lifting tasks. Further optimization, such as adaptive thresholding based on real-time force variations, could enhance the responsiveness of the device.

The user acceptance of the lifting assistance device was evaluated through questionnaires and interviews. PTs rated the direct touch feature 4.8/5, while non-experienced subjects rated it 4.3/5 on a Likert scale. These results indicate that caregivers found the system effective and intuitive to use.

An encouraging insight from PT interviews was that direct touch interaction closely resembles manual patient transfers, making the operation process feel more natural and controlled. Additionally, PTs noted that the direct touch approach allowed them to assess the care receiver’s muscle tone and body condition more effectively, which is an advantage compared to traditional lift devices with remote controllers.

Although the results validate the effectiveness of the device and the acceptance of direct touch introduced into the lifting assistance device, this study has several limitations. The first limitation is the small sample size. This study included only 12 participants, with just four PTs, which may not fully represent the variability in caregiver operation. A larger, more diverse participant pool would be necessary to strengthen statistical validation. Additionally, the study primarily relied on cable tension variations for intention detection and evaluated only the dummy’s position and rotational angle without incorporating kinematic data, interaction forces, or EMG measurements. The absence of these objective biomechanical and physiologic metrics limits the ability to fully assess system performance. Future studies should integrate these additional measurements to provide a more comprehensive evaluation of device effectiveness.

Another limitation is that the current prototype focuses only on lifting and rotating movements without incorporating translational support, such as moving a patient from a bed to a wheelchair. Future iterations of the device should incorporate the entire transfer process to expand the capability. Finally, this study used a dummy to simulate severely impaired patients, and the evaluation only reflects the caregiver’s perspective. Future work should involve trials with actual patients to assess their experience and feedback.

## 5 Conclusion

This study focused on the evaluation of a lifting assistance device designed to enhance the quality of life for both caregivers and care receivers. To achieve this goal, we proposed the concept of direct touch between caregivers and care receivers and during the lifting process. Twelve subjects, comprising four PTs and eight non-experienced individuals, tested the device’s usability and acceptance. The results demonstrate that the system could detect caregiver intentions with over 70% accuracy, regardless of their level of transfer experience. Questionnaires using a 5-point Likert scale after the experiments revealed strong acceptance of the device’s operational concept—particularly the direct touch between the caregiver and the care receiver—across both PTs and non-experienced subjects. This study underscores the significance of maintaining the human touch in the lifting process during caregiving tasks.

## Data Availability

The datasets presented in this article are not readily available because of anonymization and subject consent. Requests to access the datasets should be directed to Jiang Ming, jiang.m.889e@m.isct.ac.jp.
